# Pulse-to-pulse wavefront sensing at free-electron lasers using ptychography^1^

**DOI:** 10.1107/S1600576720006913

**Published:** 2020-07-08

**Authors:** Simone Sala, Benedikt J. Daurer, Michal Odstrcil, Flavio Capotondi, Emanuele Pedersoli, Max F. Hantke, Michele Manfredda, N. Duane Loh, Pierre Thibault, Filipe R. N. C. Maia

**Affiliations:** a Department of Physics and Astronomy, University College London, London, UK; b Department of Physics and Astronomy, University of Southampton, Southampton, UK; c Department of Cell and Molecular Biology, Uppsala University, Uppsala, Sweden; d Department of Biological Sciences, National University of Singapore, Singapore; e Paul Scherrer Institut, Villigen, Switzerland; f Elettra-Sincrotrone Trieste, Trieste, Italy; g Department of Chemistry, Oxford University, Oxford, UK; h Department of Physics, National University of Singapore, Singapore

**Keywords:** ptychography, X-ray free-electron lasers, XFELs, wavefronts, ultra-short pulses

## Abstract

The pressing need for knowledge of the detailed wavefront properties of ultra-bright and ultra-short pulses produced by free-electron lasers has spurred the development of several complementary characterization approaches. Here a method based on ptychography is presented that can retrieve high-resolution complex-valued wavefunctions of individual pulses without strong constraints on the illumination or sample object used. The technique is demonstrated within experimental conditions suited for diffraction experiments and exploiting Kirkpatrick–Baez focusing optics. This lensless technique, applicable to many other short-pulse instruments, can achieve diffraction-limited resolution.

## Abstract 2

Understanding the wavefront of ultra-bright and ultra-short pulses of X-ray free-electron lasers is both important and challenging. A method based on ptychography that can retrieve full high-resolution complex-valued wave functions of individual pulses is presented.

## 1. Introduction   

Free-electron lasers (FELs) are opening the way to a number of new research paths. Within the field of microscopy, the highly coherent and short pulses produced by FELs are used to conduct diffractive imaging of individual particles, also called flash X-ray imaging, potentially down to atomic resolution (Neutze *et al.*, 2000 ▸; Chapman *et al.*, 2006 ▸; Seibert *et al.*, 2011 ▸). Many other investigations exploit FEL tight focal spots to maximize fluence or improve spatial resolution (Willems *et al.*, 2017 ▸; Vidal *et al.*, 2017 ▸; Mincigrucci *et al.*, 2018 ▸). For all these applications, a reliable high-resolution characterization of the shot-to-shot focal spot is crucial. A number of beam diagnostics methods have been purposely designed for this task. However, with ultra-short pulses (down to femtoseconds) which can reach a flux sufficient to destroy or irreversibly damage most targets, these methods mostly provide only partial information about the wavefront, such as position, size, shape, intensity or curvature (Chalupsky *et al.*, 2011 ▸; Vartanyants *et al.*, 2011 ▸; Rutishauser *et al.*, 2012 ▸; Loh *et al.*, 2013 ▸; Sikorski *et al.*, 2015 ▸; Keitel *et al.*, 2016 ▸; Daurer *et al.*, 2017 ▸). A recent grating-based method can provide real-time wavefront distributions (Schneider *et al.*, 2018 ▸; Liu *et al.*, 2018 ▸), though away from the focal plane and with a resolution limited by the grating’s manufacturing process.

At third-generation synchrotron sources, ptychography is now a popular wavefront-characterization tool (Kewish *et al.*, 2010 ▸; Schropp *et al.*, 2010 ▸; Takahashi *et al.*, 2011 ▸; Hönig *et al.*, 2011 ▸; Vila-Comamala *et al.*, 2011 ▸), thanks to its ability to retrieve the complex-valued wavefield in or close to the focal plane (the probe) along with the transmission function of the sample (the object) (Thibault *et al.*, 2009 ▸; Maiden & Rodenburg, 2009 ▸). Further improvements to ptychographic reconstruction algorithms address additional sources of data degradation, such as partial coherence (Thibault & Menzel, 2013 ▸), scanning-position errors (Guizar-Sicairos & Fienup, 2008 ▸; Maiden *et al.*, 2012 ▸; Beckers *et al.*, 2013 ▸; Zhang *et al.*, 2013 ▸; Tripathi *et al.*, 2014 ▸) and probe variations (Odstrcil *et al.*, 2016 ▸). The recovered illumination wavefields can be numerically propagated to refine the focal position or to reveal optics-induced aberrations.

Ptychography has also been applied to characterize the wavefront at a FEL (Schropp *et al.*, 2013 ▸). That experiment relied on a constant illumination to reconstruct the object, a justified assumption given the small aperture of the focusing optics used. That assumption was later validated by refining the individual reconstructions, while assuming a constant object. The algorithm we propose does not rely on a constant illumination and allows single-pulse probes and the object to all vary at every iteration. The problem is kept constrained by representing all probes as a linear combination of the same components. Effectively reducing the constraints on the probes and the object, this method can be applied in a wider range of experimental conditions.

## 2. Experimental setup   

The experiment was carried out at the Diffraction and Projection Imaging (DiProI) beamline (Capotondi *et al.*, 2013 ▸, 2015 ▸) at FERMI, which is an extreme ultraviolet and soft X-ray seeded FEL. Using the FEL-2 line (Allaria *et al.*, 2012 ▸, 2013 ▸, 2015 ▸), 10 µJ pulses were produced at a photon energy of 83 eV, equivalent to a wavelength of 15 nm.

A test pattern (Xradia X30-30-2) consisting of a 110 nm Si_3_N_4_ membrane with a 200 nm thick Au pattern deposited on top was used as a sample. At 83 eV, the sample behaves as a binary object since the gold-plated areas absorb completely the incident X-rays. To avoid damage to the sample, a combination of attenuators reduced the beam flux by about four orders of magnitude. Solid Zr (600 nm) and Al (200 nm) attenuators were complemented with a 6 m long gas chamber filled with 2.8 × 10^−2^ mbar of N_2_. Being located tens of metres upstream of the experimental chamber, the attenuators were expected to induce negligible deformations of the wavefront, while keeping each pulse well below the sample’s damage threshold, given by an Au melting dose of 0.4 eV per atom (David *et al.*, 2011 ▸).

**Figure 1:**
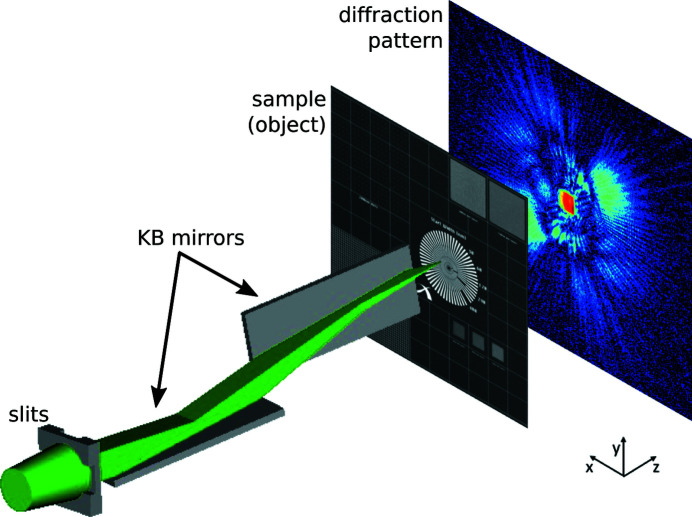
Diagram of the experimental setup. Adjustable slits form an aperture that admits the central part of the FEL beam. KB mirrors focus the beam onto a small area of the sample, which is scanned with a translation stage in the *xy* plane. The intensities of the resulting free-space-propagated exit waves (*i.e*. the diffraction patterns) are recorded by a detector downstream along *z*.

Fig. 1 ▸ gives a schematic representation of the experimental setup. A pair of perpendicular bendable Kirkpatrick–Baez (KB) mirrors focused the FEL beam down to a spot size of under 10 × 10 µm, as expected from optics specifications and previous measurements carried out at the same beamline (Raimondi *et al.*, 2013 ▸; Capotondi *et al.*, 2015 ▸). The vertical and horizontal mirrors had focal lengths of 1.75 and 1.2 m, respectively (Raimondi *et al.*, 2013 ▸). The sample was mounted on a three-axis translation stage with an encoder resolution of 100 nm which was positioned close to the focal plane and within a vacuum chamber. Far-field diffraction patterns were detected with an in-vacuum CCD camera (PI-MTE:2048B) located 150 mm downstream from the sample and featuring 2048 × 2048 pixels, 13.5 µm each. To decrease readout time to 2 s, only the intensities collected by the central 1000 × 1000 pixels were recorded. As the readout frequency was lower than the FEL’s 10 Hz repetition rate, a fast shutter was used to prevent more than one pulse from contributing to each detector reading. Because of further overhead, the acquisition rate was effectively reduced to 0.2 Hz, *i.e.* only one every 50 FEL pulses was recorded.

## 3. Data acquisition and correction   

**Figure 2:**
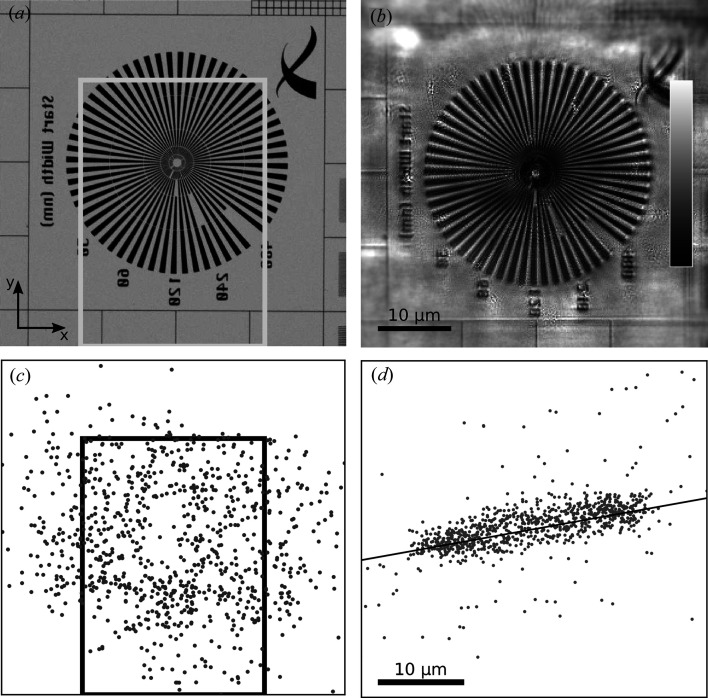
(*a*) SEM image of the Siemens star test pattern; the scale bar is in (*b*). (*b*) Amplitude of the ptychographic reconstruction; the color bar represents transmission between 0 and 1. (*c*) Probe positions recovered via our position-correction algorithm; the scale bar is in (*b*). The area covered by the motor positions used for the ptychographic scan is indicated with rectangles in (*a*) and (*c*). (*d*) Position correction for each scanning point; null correction (0, 0) at the center. The main axis of this two-dimensional correction distribution is indicated, revealing a dominant vibration component along the *x* axis.

The ptychographic data were collected by scanning an area of the sample which included a 30 × 30 µm Siemens star whose scanning electron microscopy (SEM) image is represented in Fig. 2 ▸(*a*). Three 25 × 25 µm spiral scans in the *xy* plane were used, with a step size of 2.5 µm for a total of 101 positions each. Five single-pulse diffraction patterns were collected at each scanning position, for a total of 1515 frames over the three spiral scans. The center of the last spiral scan was translated in *y* to extend the overall scanned area, which is highlighted by the rectangles in Figs. 2 ▸(*a*) and 2 ▸(*c*). The collected diffraction patterns were binned by a factor of two and then padded to a size of 512 × 512 pixels in order to decrease computational cost. From each frame, a dark frame was subtracted which was obtained by averaging 100 frames recorded with the beam shutter closed. Detector counts were thresholded to a value of 0 in order to remove unphysical negative counts and converted into units of photon counts.

A large instability in the photon-beam position on the sample was found to have occurred throughout data acquisition and was caused by vibrations affecting an upstream optical component. The instability was of such a large amplitude – tens of micrometres – that the scanning positions recorded by the sample motors could not be used without corrections. Most known position-refinement approaches within ptychographic algorithms (Guizar-Sicairos & Fienup, 2008 ▸; Maiden *et al.*, 2012 ▸; Beckers *et al.*, 2013 ▸; Zhang *et al.*, 2013 ▸; Tripathi *et al.*, 2014 ▸) have been designed to account for minor deviations from the expected positions, typically smaller than the scanning step size. Here, a customized approach was implemented to accommodate such a large instability. Coarse positions were first obtained by cross correlating the recorded diffraction patterns with simulations based on the interaction of a simulated probe and an object modeled from the available high-resolution image of the test pattern [*cf*. Fig. 2(*a*)]. The coarse positions were then further refined with a purposely designed position-refinement algorithm (Odstrčil *et al.*, 2018 ▸). Diffraction patterns corresponding to positions far from the intended field of view were discarded, as were others for which position refinement failed, effectively reducing the data set to 937 frames.

Fig. 2 ▸(*c*) shows the corrected and refined positions, along with the intended rectangular scanning area. The deviation of the retrieved scanning positions from the nominal ones is represented in Fig. 2(*d*), where the main axis of the vibration distribution is indicated, revealing a dominant horizontal component associated with a standard deviation of 9.2 µm.

Although the coarse position correction was only possible thanks to the in-depth characterization of the test pattern prior to the experiment, the developed position-refinement algorithm is expected to benefit several other experiments performed at FELs that are affected by intrinsic pointing instability, besides minor vibrations of the optics and sample stages.

## 4. Ptychographic reconstruction   

For the ptychographic reconstruction of single-pulse wavefronts, the collected frames associated with the corrected positions were used within an algorithm derived from orthogonal probe relaxation ptychography (OPRP) (Odstrcil *et al.*, 2016 ▸). This approach recovers a different probe *P*_*j*_ for each of the *N* diffraction patterns *I*_*j*_ – with the frame index *j* varying between 1 and *N* – while keeping the problem over-constrained through dimensionality reduction. This is achieved by adding a singular-value decomposition (SVD) step at the end of every iteration of the ptychographic reconstruction algorithm which generates the probes’ main principal components. Individual probes are thus modeled as linear combinations of a number of dominant components (also called ‘modes’ or ‘eigen­probes’) recovered dynamically and without *a priori* information.

Given the complex matrix *P* whose columns contain estimates of the individual probes *P*_*j*_, applying SVD to *P* leads to *P* = *U*Σ*V* 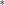, where *V* 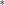 denotes the Hermitian transposition of *V* and both *U* and *V* are unitary matrices, such that *UU* 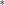 = *U* 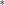 *U* = *I* and *V* 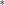 *V* = *VV* 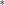 = *I*, with *I* as the identity matrix. By multiplying *P* with its Hermitian transpose *P* 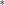, we obtain
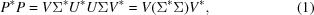
with *P* 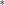 *P* as a Hermitian matrix and Σ
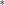
Σ as a diagonal matrix. This is equivalent to the eigenvalue problem 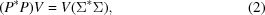
so that the nonzero elements on the diagonal of Σ correspond to the square roots of the eigenvalues of *P* 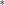 *P*.

The solution to this problem within the ptychographic algorithm is implemented as a truncated diagonalization: the *N* eigenvalues and main *k* eigenvectors 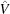 can be retrieved, with *k* < *N* and 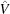 denoting truncation of *V*. Applying this in the SVD step, a set of *k* orthogonal components *M* is generated via 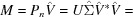 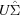, where *P*_*n*_ denotes the probe matrix *P* at the *n*th iteration. The obtained component matrix *M* is then used to generate the updated probes: 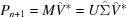.

As usual for ptychography, the object’s transmission function is also retrieved without enforcing any prior knowledge. The essence of OPRP is compatible with any ptychographic reconstruction algorithm and has been implemented within the *PtyPy* reconstruction suite (Enders & Thibault, 2016 ▸) for both difference-map (DM) (Thibault *et al.*, 2009 ▸) and maximum-likelihood (ML) (Thibault & Guizar-Sicairos, 2012 ▸) algorithms. The reconstructions presented here were obtained using DM followed by ML refinement, both using a ten-component decomposition of the retrieved probes. The number of components *k* = 10 was optimized empirically by observing that the magnitude of singular values dropped fast for higher components within reconstructions with *k* > 10. This indicated that the contribution of higher components was no longer significant for the reconstruction.

Given the experiment geometry and detector specifications, the achieved pixel size in the ptychographic reconstruction was 162 nm. The initial illumination function used for all probes was produced by numerical back-propagation of the mean diffraction pattern. The initial object was assigned a uniform unit transmission function, *i.e.* the algorithm started with no assumptions on the object. Both the object and all probes were allowed to vary at every iteration of the ptychographic algorithm, which ran 200 iterations of DM followed by 800 iterations of ML refinement.

## 5. Results   

The absolute value of the retrieved object transmission function is represented in Fig. 2 ▸(*b*), which can be compared with the SEM image of the same region in Fig. 2 ▸(*a*). The agreement between the two images is apparent and features of the Au test pattern can be resolved down to a size of 195 nm. The significant beam instability had mainly two effects. On the one hand, it caused some of the scanned areas to be only sparsely sampled with respect to the intended overlap, which is known to induce artefacts. On the other hand, it led to a larger area of the sample being imaged, *i.e*. outside the field of view of the original scanning area. The combination of these two effects can be observed on the Xradia logo, for example, which lies outside the intended field of view and is reconstructed nevertheless, though with artefacts.

**Figure 3:**
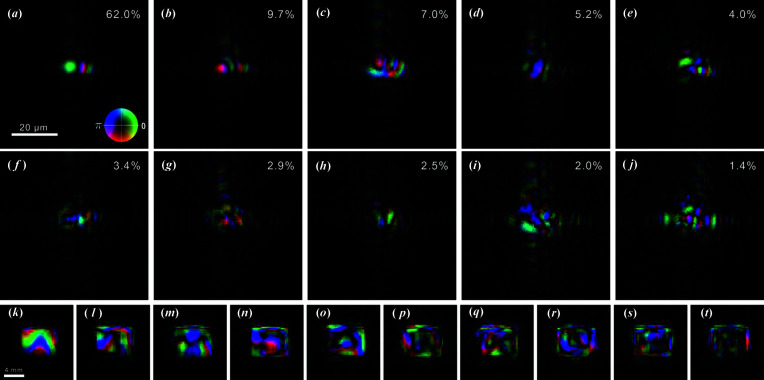
(*a*)–(*j*) Ten components of reconstructed probes obtained via OPRP reconstruction. Singular values are annotated on each component. (*k*)–(*t*) Back-propagation of the same components to the virtual secondary source plane, neglecting the spherical wave term. Amplitude is mapped to brightness and phase is mapped to hue according to the color wheel in (*a*); the brightness scale is such that its maximum has been adjusted for every frame individually to match its brightest pixel.

The fidelity of the reconstructed object confirms the robustness of the algorithm and the validity of the retrieved probes, whose components are represented in Figs. 3 ▸(*a*)–3 ▸(*j*). Though each component’s contribution to each pulse varies, a qualitative indication of their relative weight is given by the singular values obtained through truncated SVD and annotated on each component [*cf.* Figs. 3 ▸(*a*)–3 ▸(*j*)]. To improve readability, these values have been rescaled so that their sum equals unity and are displayed as percentages. When back-propagated to the virtual secondary source plane located at the mid-point between the pair of KB mirrors, 1.48 m upstream from the interaction plane [Figs. 3 ▸(*k*)–3 ▸(*t*)], the components exhibit the expected intensity distribution, with the intensity dropping to negligible values outside of the secondary source. The virtual secondary source appears rectangular because of the finite size of the KB mirrors, which at grazing incidence effectively act as rectangular apertures.

**Figure 4:**
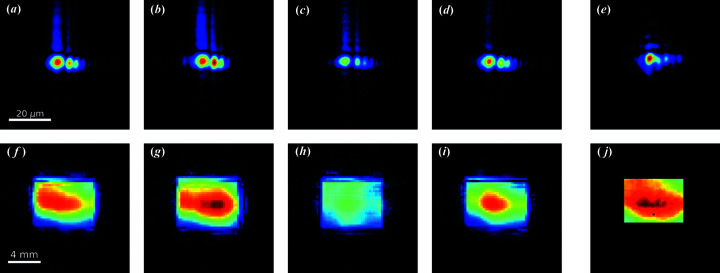
(*a*)–(*d*) Amplitude of four of the *N* = 937 retrieved probes obtained via OPRP reconstruction. (*e*) Amplitude of a probe retrieved from Hartmann sensor data. (*f*)–(*j*) Back-propagation of (*a*)–(*e*) to the virtual secondary source plane, neglecting the spherical wave term. Color scales are shared among (*a*)–(*e*) and among (*f*)–(*j*), revealing intensity variations.

The amplitude of four selected probes is shown in Figs. 4 ▸(*a*)–4 ▸(*d*) as a representative sample of the full stack of *N* = 937 retrieved probes, which is available as a video in the supporting information. For comparison purposes, the amplitude of a probe retrieved starting from Hartmann sensor data is shown in Fig. 4 ▸(*e*). The Hartmann sensor routinely available at the beamline for wavefront sensing (Raimondi *et al.*, 2013 ▸) was operated within the same experimental conditions as those of the ptychography experiment, although it did not sample the same pulses. Figs. 4 ▸(*f*)–4 ▸(*j*) show the amplitudes of the same wavefronts [Figs. 4 ▸(*a*)–4 ▸(*e*)] after they have been numerically back-propagated to the virtual secondary source plane. Figs. 4 ▸(*a*)–4 ▸(*e*) share the same color scaling, as do Figs. 4 ▸(*f*)–4 ▸(*j*). This highlights the presence of overall intensity variation between different wavefronts. Pulse-to-pulse variations can also be observed as variations of the photon spatial distribution both at the sample plane and at the virtual secondary source plane.

Both the wavefronts recovered via ptychographic reconstruction and that originating from Hartmann sensor data reveal diffraction effects caused by the KB optical figure error and – to a lesser extent – by the interaction of the coherent FEL beam and the effective rectangular secondary source. These effects were also found within other independent wavefront characterization experiments carried out at the same FEL and beamline (Capotondi *et al.*, 2015 ▸; Schneider *et al.*, 2018 ▸; Raimondi *et al.*, 2019 ▸; Manfredda *et al.*, 2019 ▸).

**Figure 5:**
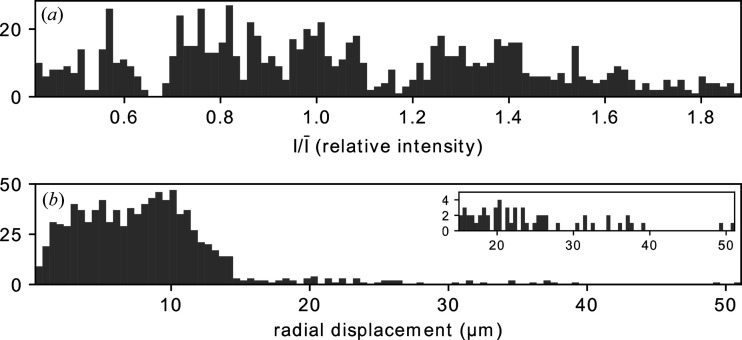
Histograms of (*a*) the intensity of each probe relative to the median intensity and (*b*) the radial displacement of the center of mass of each probe relative to the center of the detector. Inset in (*b*) is a rescaled version of a portion of the same histogram.

Using the full stack of retrieved probes, it is possible to gather valuable statistical information, such as variations in intensity and beam pointing. Fig. 5 ▸(*a*) illustrates the fluctuations of the total intensity found for each probe relative to the median total intensity, revealing significant variations, with a relative standard deviation of 0.4. Fig. 5 ▸(*b*) shows the radial displacement of the center of mass of each probe relative to the center of the detector, revealing a median relative dis­place­ment of 8 µm, which confirms the presence of beam-pointing variations.

**Figure 6:**
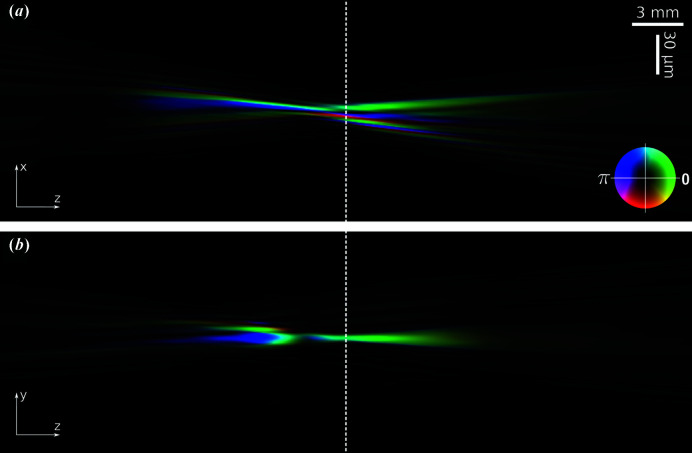
Horizontal (*a*) and vertical (*b*) sections of ptychographic reconstruction of the main component propagated around the focal position (±20 mm). Image scaling is different in its two dimensions according to the scale bars in (*a*). Amplitude is mapped to brightness and phase is mapped to hue according to the color wheel in (*a*).

We numerically propagated the main component – as it is the most representative part of all retrieved probes – downstream and upstream from the object plane to investigate the region around the focal position. Sections of the propagated main component are shown in Fig. 6 ▸ and can be compared with the unpropagated reconstructed main component from Fig. 3 ▸(*a*). The dotted line in Fig. 6 ▸ indicates the sample plane; the focal plane has been estimated to be some 2 mm further upstream, as the plane at which the beam size is minimized.

There exist a few key differences between ptychographic wavefront characterization and grating-based methods (Schneider *et al.*, 2018 ▸; Liu *et al.*, 2018 ▸). The latter can be used for online characterization and, for instance, can provide information on the same wavefronts used for imaging experiments. However, they are limited by the grating manufacturing process, particularly at higher energies. Also, they recover information far from the focal position and tend to underestimate the intensity in the tails of the power distribution as well as being insensitive to beam-position variations. For these reasons, grating-based and lensless wavefront characterization could be considered complementary approaches and combined within the same experiments.

Furthermore, pulse prediction using machine learning has been revealed as another valuable tool for FEL experiments (Sanchez-Gonzalez *et al.*, 2017 ▸). However, its successful application relies on the availability of training data sets with in-depth information on single-pulse wavefronts. We envision that our ptychographic approach could be used to produce such training data sets before imaging experiments and then be combined with high-repetition-rate diagnostics to predict single-pulse properties from fewer and simpler parameters collected during the experiment.

## 6. Conclusions   

We have demonstrated pulse-to-pulse ptychographic wavefront reconstruction for a FEL instrument without assuming a constant illumination.

Unlike previous ptychographic methods, OPRP allows all single-pulse probes and the object to vary simultaneously. It does not rely on strong constraints on the object to recover single-pulse information and can start from a random initial guess for the object and reconstruct it without enforcing any assumption on it. Our approach provides the complex-valued wavefront of each pulse directly at the sample plane, in its near-focus position. As a lensless microscopy technique, its achievable resolution is limited only by flux and detector numerical aperture.

The full set of 937 probes retrieved with our OPRP approach reveals pulse-to-pulse variations, providing statistical information on FEL beam fluctuations and direct insight into FERMI’s performance. The retrieved probes also confirm the elongated shape of the beam at and around the focal position, as observed in other experiments carried out at the same beamline previously (Raimondi *et al.*, 2013 ▸; Capotondi *et al.*, 2015 ▸) and since (Schneider *et al.*, 2018 ▸; Raimondi *et al.*, 2019 ▸).

The application of pulse-to-pulse ptychography can also be extended to other imaging experiments that would benefit from the relaxation of the single-illumination probe constraint, for example at laboratory sources. Here, we have demonstrated its application for wavefront characterization experiments at FELs, contributing to the development of FELs and FEL-based science.

## Supplementary Material

Supporting video - the full stack of 937 retrieved probes. DOI: 10.1107/S1600576720006913/zy5004sup1.mp4
